# A Review of the Development of Biopolymer Hydrogel-Based Scaffold Materials for Drug Delivery and Tissue Engineering Applications

**DOI:** 10.3390/gels11030178

**Published:** 2025-03-01

**Authors:** Madhappan Santhamoorthy, Seong-Cheol Kim

**Affiliations:** School of Chemical Engineering, Yeungnam University, Gyeongsan 38541, Gyeongbuk, Republic of Korea

**Keywords:** biopolymers, hydrogels, scaffold material, drug delivery, tissue engineering, wound healing

## Abstract

Biopolymer hydrogel-based scaffold materials have received a lot of interest in tissue engineering and regenerative medicine because of their unique characteristics, which include biocompatibility, biodegradability, and the ability to replicate the natural extracellular matrix (ECM). These hydrogels are three-dimensional biopolymer networks that are highly hydrated and provide a supportive, wet environment conducive to cell growth, migration, and differentiation. They are especially useful in applications involving wound healing, cartilage, bone, and soft tissue regeneration. Natural biopolymers such as collagen, chitosan, hyaluronic acid, and alginate are frequently employed as the foundation for hydrogel fabrication, providing benefits such as low toxicity and improved cell adherence. Despite their potential, biopolymer hydrogel scaffolds have various difficulties that prevent broad clinical implementation. Key difficulties include the challenge of balancing mechanical strength and flexibility to meet the needs of various tissues, managing degradation rates to line up with tissue regeneration, and assuring large-scale manufacturing while retaining scaffold uniformity and quality. Furthermore, fostering appropriate vascularization and cell infiltration in larger tissues remains a significant challenge for optimal tissue integration and function. Future developments in biopolymer hydrogel-based scaffolds are likely to concentrate on addressing these obstacles. Strategies such as the creation of hybrid hydrogels that combine natural and synthetic materials, smart hydrogels with stimulus-responsive features, and 3D bioprinting technologies for accurate scaffold production show significant potential. Furthermore, integrating bioactive compounds and growth factors into hydrogel matrices to promote tissue regeneration is critical for enhancing therapeutic results.

## 1. Introduction

Biopolymer hydrogel-based scaffolds have emerged as a game-changing class of materials in biomedicine, notably for drug delivery and tissue engineering applications. These hydrogels, made from hydrophilic polymer networks, have extraordinary water retention properties and closely resemble the extracellular matrix (ECM) of real tissues. Biopolymers produced from natural sources, such as proteins (e.g., collagen, gelatin, silk fibroin) and polysaccharides (e.g., chitosan, alginate, hyaluronic acid), are particularly valued for their intrinsic biocompatibility, biodegradability, and ability to promote biological functions [1,2].

Recent advances have accelerated the functionalization and use of biopolymer hydrogel scaffolds. In tissue engineering, scaffolds provide structural support and bioactive environments that promote cell adhesion, proliferation, and differentiation, allowing injured tissues to regenerate [3]. In drug delivery, biopolymer hydrogels serve as carriers for therapeutic drugs, allowing for targeted, controlled, and prolonged drug release. These qualities are crucial for increasing therapy effectiveness while reducing systemic adverse effects. Researchers have developed unique techniques to alleviate typical hydrogels’ drawbacks, including poor mechanical characteristics and controllable disintegration rates [4,5]. Strategies such as synthetic polymers, nanomaterials, or crosslinking agents can improve the mechanical strength and tunability of biopolymer hydrogels. Furthermore, advances in manufacturing techniques, like 3D bioprinting, have made it possible to create sophisticated, biomimetic structures with precise spatial organization of cells and bioactive components.

The development of stimulus-responsive hydrogels that may react to environmental stimuli such as pH, temperature, or enzyme activity has increased their value. These smart hydrogels enable on-demand, spatiotemporal drug release, bringing a new level of precision to therapy. Composite hydrogels, which combine various biopolymers and other materials, have also demonstrated potential for improving scaffold adaptability and applicability [6,7]. This review focuses on current advances in biopolymer hydrogel-based scaffolds, emphasizing their innovations, uses, and prospects for influencing the future of drug delivery and tissue engineering.

## 2. Properties and Advantages of Biopolymer Hydrogels

Biopolymer hydrogel-based scaffold materials have several benefits, making them essential for advances in tissue engineering and regenerative medicine. These materials, derived from natural polymers such as alginate, chitosan, gelatin, and hyaluronic acid, are intrinsically biocompatible and biodegradable, allowing for safe integration with biological systems while lowering long-term toxicity hazards [1]. Their wet, porous shape resembles the ECM, creating an ideal environment for cell adhesion, proliferation, and differentiation.

**Figure 1:**
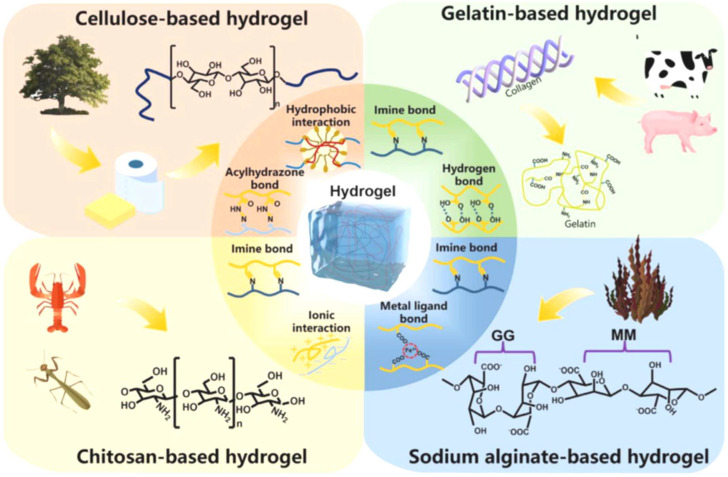
The main interactions of hydrogels from different biopolymers. Adapted with permission from Ref. [9]. Copyright 2023, Elsevier.

One significant benefit is their customizable mechanical and physical qualities, which enable customization for particular applications like bone, cartilage, and soft tissue regeneration. Biopolymer hydrogels also promote nutrition and waste exchange, which is critical for cell survival. They can also be functionalized with bioactive compounds, growth factors, or medicines to improve treatment results and direct tissue healing processes. Their simplicity of processing and adaptation to modern manufacturing processes, such as 3D bioprinting, reinforces their status as flexible and effective scaffold materials in biomedical applications [8]. The dynamic nature of these interactions enables the network structure of hydrogels to regenerate upon fracture, giving them self-healing characteristics. Biopolymer-based hydrogels are often made from gelatin, chitosan, sodium alginate, cellulose, or their derivatives (Figure 1) [9].

### 2.1. Biocompatibility of Biopolymer Hydrogels

Biopolymer hydrogel-based scaffold materials are well known for their high biocompatibility, which is essential for applications in tissue engineering, drug delivery, and regenerative medicine. Biopolymers including alginate, chitosan, gelatin, and hyaluronic acid are obtained from natural sources (ECM) of living tissues. This structural similarity promotes cell adhesion, proliferation, and differentiation, resulting in successful integration into host tissues while avoiding detrimental immunological reactions [4].

The hydrophilic characteristic of biopolymer hydrogels improves their capacity to retain water and nutrients, resulting in a cellular-friendly milieu that promotes metabolic activity. Their intrinsic biodegradability guarantees that the scaffold degrades into non-toxic byproducts, which aligns with the body’s natural healing processes and eliminates the need for surgical removal. Furthermore, biopolymer hydrogels may be created to include bioactive compounds like growth factors, peptides, or drugs, which improve therapeutic efficacy and promote certain cellular behaviors [10].

**Figure 2:**
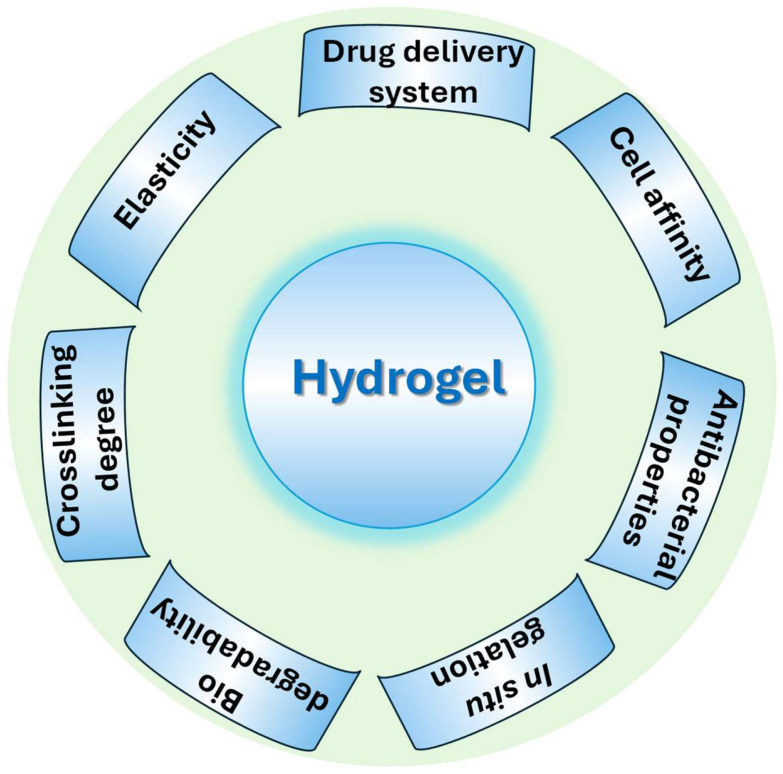
Basic requirements for an ideal hydrogel material.

Biocompatibility is also determined by the construction procedures and crosslinking tactics utilized, which can affect the mechanical characteristics and degradation rates of scaffolds. Advances in these approaches have enabled the development of hydrogels with precise pore architectures and mechanical strengths customized to certain tissue types [11]. As a result, biopolymer hydrogel-based scaffolds are emerging as a key component in the creation of next-generation biomaterials for therapeutic use. The three-dimensional network architecture of hydrogels may be easily changed to produce the necessary physical qualities, with physical metrics like crosslinking degree, elastic modulus, and degradation rate serving as indications. Thus, shape and flexibility may conform to the target tissue, and the degradation time can be adjusted to coincide with the progression of tissue regeneration (Figure 2).

### 2.2. Biodegradability of Biopolymer Hydrogels

The biodegradability of biopolymer hydrogel-based scaffold materials is a distinguishing feature that increases their applicability in biomedical and environmental settings. These scaffolds, made from natural polymers such as alginate, chitosan, collagen, and hyaluronic acid, are intended to break down into non-toxic byproducts by natural enzymatic or hydrolytic processes [12]. This property is especially useful in tissue engineering, where the scaffold slowly disintegrates as new tissue grows, eliminating the need for surgical removal and reducing long-term consequences.

The breakdown rate of biopolymer hydrogels may be accurately regulated by adjusting their crosslinking density and polymer composition or adding additives. This adaptability enables them to suit the unique requirements of diverse applications, such as rapid degradation for drug delivery systems or slower rates for long-term tissue support [13]. The body safely metabolizes or excretes degradation byproducts, which are frequently water-soluble and bioresorbable. Biodegradability also supports environmental sustainability goals. In contrast to manufactured, non-biodegradable polymers, these materials disintegrate naturally, lowering their environmental impact [14]. Furthermore, the capacity to mix biopolymers with other biodegradable materials broadens their functional capabilities.

### 2.3. Hydrophilicity of Biopolymer Hydrogels

The hydrophilicity of biopolymer hydrogel-based scaffold materials is an important characteristic that improves their performance in biomedical and tissue engineering applications. Biocompatible hydrogels are made up of natural polymers such as alginate, chitosan, gelatin, and hyaluronic acid and have a highly water-absorbing structure due to the availability of hydrophilic functional groups such as hydroxyl, carboxyl, and amine groups in their molecular chains [15]. This hydrophilicity allows the scaffolds to retain substantial amounts of water while retaining structural integrity, resulting in a moist and porous environment that is similar to the ECM. This characteristic is critical for increasing cell adhesion, proliferation, and migration, as well as enabling nutrition and oxygen flow to cells implanted in the scaffold. Furthermore, the hydrated state of these scaffolds reduces friction and irritation during implantation, boosting biocompatibility [16].

Hydrophilicity is particularly important in drug delivery applications because it allows for the integration and prolonged release of therapeutic substances, resulting in a more regulated and targeted treatment strategy [17] (Table 1). Furthermore, the ability to control the degree of hydrophilicity via chemical or physical alterations provides personalized solutions for a variety of applications, including wound healing and cartilage regeneration.

## 3. Tunable Physical and Chemical Properties of Biopolymer Hydrogels

One of the primary benefits of biopolymer hydrogel-based scaffold materials is their customizable physical and chemical characteristics, which enable customization to meet particular needs in tissue engineering, drug delivery, and regenerative medicine. Physical characteristics such as mechanical strength, swelling behavior, and porosity may be adjusted by varying the polymer content, crosslinking density, and external environmental conditions [27]. This flexibility enables the development of scaffolds with varied stiffness and elasticity, which is critical for replicating the mechanical properties of various tissues such as bone, cartilage, and soft tissue.

Chemically, biopolymer hydrogels can be functionalized with bioactive molecules like growth factors, peptides, or drugs to improve particular cellular interactions and facilitate tissue healing. The addition of these bioactive substances can impact cellular activities such as adhesion, migration, and differentiation, therefore directing tissue regeneration [28]. Furthermore, the breakdown rate of these hydrogels may be adjusted by varying the polymer composition and crosslinking techniques, allowing the scaffold to deteriorate at a rate consistent with tissue development and healing processes [17]. These adjustable features also include the gel’s hydrophilicity, charge density, and sensitivity to external stimuli including pH, temperature, and ionic strength [29]. This level of control renders biopolymer hydrogel-based scaffolds flexible and suitable for a wide range of biomedical applications.

The characteristics of various natural polymers and their hydrogels can be described as follows. Natural polymers are obtained from renewable sources and display a wide range of characteristics, making them useful in a variety of applications. Polysaccharides, such as cellulose, chitosan, and alginate, are biocompatible, biodegradable, and hydrophilic, making them suitable for biomedical and packaging applications. Proteins such as gelatin and silk fibroin give mechanical strength and flexibility and are often employed in wound healing and tissue engineering. Polyesters, such as polyhydroxyalkanoates (PHAs), are microbially produced, biodegradable, and appropriate for medical sutures and drug administration. Rubbers, like natural latex, are extremely elastic and robust and are commonly employed in hemostatic agents and medical equipment. These polymers have distinct features due to their molecular structure and origin.

Hydrogels made from natural polymers are hydrophilic, three-dimensional networks that can absorb and hold significant amounts of water. They are usually biodegradable, biocompatible, and non-toxic, making them suitable for biomedical applications such as wound healing, delivery of drugs, and tissue engineering. Polysaccharide-based hydrogels, such as alginate, chitosan, and hyaluronic acid, have outstanding gelation characteristics and bioactivity. Protein-based hydrogels, such as gelatin and silk fibroin, provide adjustable mechanical strength and cell adhesion. These hydrogels show stimulus-responsive behavior, such as swelling or degrading in response to pH, temperature, or enzymes, making them ideal for controlled drug release and regenerative medicine applications.

The similarities and differences between various polymers derived from natural materials and ECMs are described as follows. Natural polymers and the extracellular matrix (ECM) have various characteristics, most notably their biocompatibility, biodegradability, and significance in supporting biological processes. Both offer structural integrity, hydration, and cell adhesion, making them ideal for biological applications. Polysaccharides (e.g., chitosan, alginate) and proteins (e.g., gelatin, silk fibroin) resemble ECM components such as glycosaminoglycans and collagen. However, the ECM is a complex, dynamic network composed of endogenous proteins, glycoproteins, and polysaccharides that actively regulate cell activity and signaling. In contrast, natural polymers, while ECM-like, may require chemical modifications or crosslinking to completely reproduce ECM functioning in tissue engineering and regenerative medicine.

## 4. Fabrication Techniques of Biopolymer Hydrogels

Biopolymer hydrogel scaffolds are developed using advanced manufacturing processes that provide exact control over their characteristics. Crosslinking is an important step in the production of biopolymer hydrogel-based scaffold materials since it has a direct impact on the scaffold’s mechanical characteristics, stability, and biodegradability [30]. The development of covalent or non-covalent connections between polymer chains improves hydrogel’s structural integrity and effectiveness in biomedical applications such as tissue engineering, drug delivery, and wound healing.

There are two main types of crosslinking methods: chemical crosslinking and physical crosslinking.

### 4.1. Chemical Crosslinking Method

The chemical crosslinking approach is commonly used to improve the structural integrity, mechanical characteristics, and biodegradability of hydrogels for biomedical purposes. Chemical crosslinking is the process of creating covalent links between polymer chains, which results in a network structure that gives stability and strength while also providing exact control over the hydrogel’s physical and chemical characteristics [31,32].

The procedure begins with the selection of biopolymers such as alginate, chitosan, collagen, or gelatin, which are inherently biocompatible and biodegradable. To begin crosslinking, crosslinking agents such as glutaraldehyde, genipin, or carbodiimides are used to establish covalent connections between polymer chains [33]. The degree of crosslinking may be adjusted by varying the quantity of the crosslinking agent and the reaction time, which determines the hydrogel scaffold’s excellent qualities.

**Figure 3:**
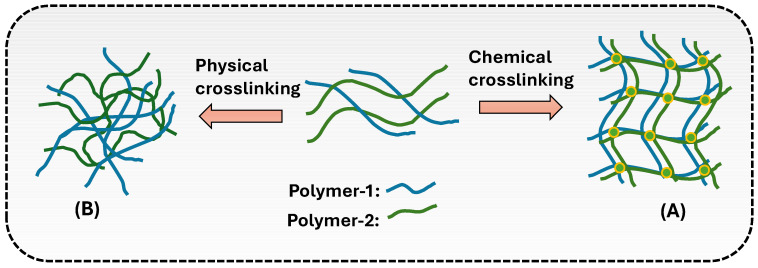
(**A**) Chemical, and (**B**) physical and crosslinking of polymers in hydrogel formation.

One of the most significant advantages of chemical crosslinking is its ability to add regulated mechanical characteristics to the hydrogel. By adjusting the crosslinking density, scaffolds with appropriate stiffness, tensile strength, and elasticity may be created, which are critical for replicating the mechanical characteristics of diverse tissues such as cartilage, bone, or soft tissues. Additionally, the hydrogel’s swelling behavior and water retention capacity may be adjusted, allowing for improved nutrition and gas exchange, which is critical for cell viability [34]. The scaffold’s breakdown rate can also be regulated by varying the degree of crosslinking. A higher crosslinking density often leads to a slower breakdown rate, which can help to maintain tissue development over time. Conversely, a lower degree of crosslinking might allow for quicker breakdown, which corresponds to the development of new tissue [35]. Chemical crosslinking can also include bioactive compounds like growth factors or peptides into the hydrogel matrix, which improves cellular connections and promotes tissue repair. This can be accomplished by functionalizing polymer chains with reactive groups that allow for the covalent attachment of bioactive molecules during the crosslinking process (Figure 3) [36].

### 4.2. Physical Crosslinking Method

The physical crosslinking approach is used to create biopolymer hydrogel-based scaffold materials by forming a three-dimensional network through non-covalent interactions such as hydrogen bonding, ionic interactions, and van der Waals forces. Unlike chemical crosslinking, which is based on covalent bonds, physical crosslinking provides reversible and dynamic interactions that are affected by external environmental elements such as temperature, pH, ionic strength, and electric fields [37]. Physical crosslinking is very useful for producing scaffolds that can respond to stimuli, making it excellent for applications such as tissue engineering, controlled drug delivery, and wound healing.

In the manufacturing process, biopolymers such as alginate, chitosan, gelatin, or hyaluronic acid are dissolved in a suitable solvent to create a gel precursor solution. Crosslinking is triggered by changing environmental parameters, such as the temperature, ionic concentration, or pH of the solution. In alginate-based hydrogels, ionic gelation involves introducing divalent cations such as calcium (Ca^2+^) to generate ionic connections between the carboxyl groups of alginate molecules, resulting in a gel network [38]. Similarly, thermoreversible hydrogels based on gelatin or agarose undergo sol-to-gel transitions when cooled, with gelation occurring as the temperature decreases.

The reversibility of physical crosslinking is a significant benefit since it enables dynamic adjustment of scaffold parameters. For example, thermoresponsive hydrogels can gel at body temperature, making them suitable for injectable or minimally invasive applications [39]. Furthermore, physical crosslinking can help to release encapsulated therapeutic substances or cells in response to external stimuli, resulting in regulated and localized medication administration. Physical hydrogels’ porosity and swelling behavior may also be regulated by varying crosslinking conditions, which is critical for building scaffolds that imitate the extracellular matrix (ECM) and stimulate tissue regeneration. Furthermore, physical and chemical crosslinking approaches can be coupled to improve the structural stability and usefulness of hydrogels, resulting in a greater variety of customized features. Wang et al. [40] prepared a dynamic crosslinked injectable hydrogel (DACS hydrogel) by mixing dopamine-grafted oxidized hyaluronic acid (DAHA) and CMCS solution based on Schiff base reactions. By this method, the authors obtained a mild, safe, and efficient gelling method. The polymers are crosslinked by Schiff base reaction and hydrogen bonding interactions.

**Figure 4:**
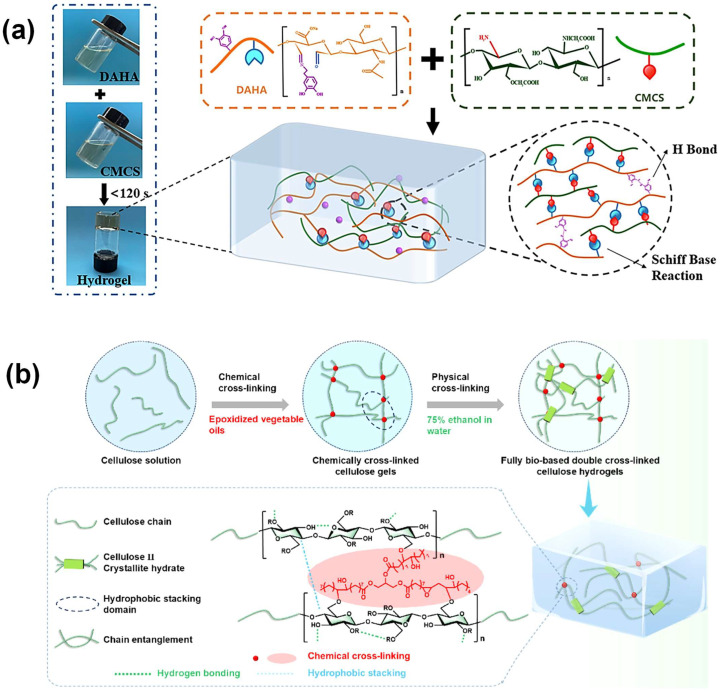
(**a**) The crosslinking of DAHA and CMC solution to form DACS hydrogels. Adapted with permission from Ref. [40]. Copyright 2023, MDPI. (**b**) Schematic diagram of the preparation process of obtaining the FBDC cellulose hydrogels. Adapted with permission from Ref. [41]. Copyright 2024, American Chemical Society.

The reaction of amine groups in the CMCS with aldehyde groups resulted in the formation of imine bonds to form covalent bonds followed by hydrogen bonding interactions between phenolic hydroxyl groups of grafted diamines (Figure 4a). Wang et al. [41] created FBDC cellulose hydrogels with great mechanical strength and toughness. They used epoxy vegetable oils as a crosslinking agent and a 75% ethanol solution. The effects of different types and molar ratios of epoxidized vegetable oil to cellulose on the properties of FBDC were investigated. The introduction of epoxidized vegetable oil could form soft domains through covalent crosslinking to withstand large deformations, while hydrogen bonding between cellulose chains and chain entanglement effectively disperses stress. For the flexible wearable power supply component, an FBDC hydrogel electrolyte with good mechanical qualities (maximum tensile stress of 6.1 MPa, elongation at break of 290%), high ionic conductivity (35.35 mS cm^−1^), and a stable electrochemical window (2.4 V) was achieved (Figure 4b).

### 4.3. Three-Dimensional (3D) Printing Method

The 3D printing technique for producing biopolymer hydrogel-based scaffold materials has transformed tissue engineering and regenerative medicine by allowing the fabrication of extremely accurate, customized, and sophisticated scaffold designs. This approach enables the creation of scaffolds with complicated geometry that mimic the natural ECM, resulting in an ideal environment for cell adhesion, proliferation, and tissue regeneration [42].

In 3D printing, biopolymer hydrogel inks are created by dissolving natural polymers like alginate, chitosan, gelatin, hyaluronic acid, or collagen in suitable solvents. These hydrogel inks are then extruded through a printer to create the scaffold layer by layer [43,44]. The fundamental problem in 3D printing hydrogels is ensuring that the material has the viscosity and mechanical stability to print while also allowing for gelation to preserve its form. To achieve this, the hydrogels might be physically or chemically crosslinked during or after printing [45].

Ionic crosslinking is commonly employed with alginate-based inks. The hydrogel is crosslinked by adding divalent ions, such as calcium (Ca^2+^), during printing.

**Figure 5:**
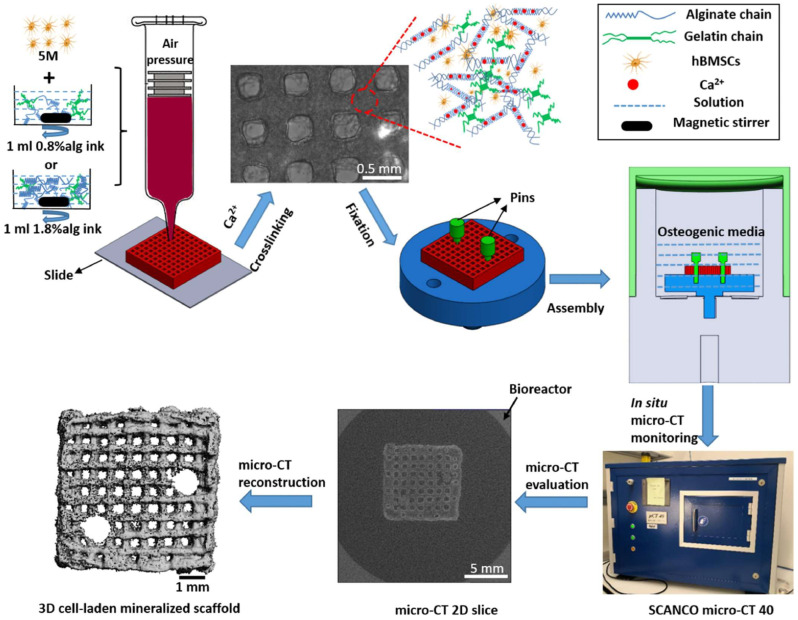
Bioink preparation with different alginate concentrations, 3D bioprinting, crosslinking, bioreactor assembling, time-lapsed micro-CT monitoring, micro-CT evaluation, and micro-CT reconstruction processes. Adapted with permission from Ref. [50]. Copyright 2020, Elsevier.

This allows the hydrogel to create a stable structure while maintaining its porosity and water retention capabilities [46,47]. Thermosensitive hydrogels (such as gelatin) can gel when cooled, allowing the hydrogel to harden at body temperature following extrusion. Three-dimensional printing has the benefit of being able to produce very complex scaffolds with controlled pore diameters, linked channels, and mechanical property gradients that replicate the structural and functional features of natural tissues [48,49]. Furthermore, 3D-printed scaffolds may contain cells, growth factors, and other bioactive substances directly due to the printing process, enhancing tissue regeneration and allowing for regulated drug distribution. This layer-by-layer technique provides the precision required to produce scaffolds with the optimal structural and biochemical characteristics for specific tissue types, such as bone, cartilage, or soft tissues (Figure 5) [50].

The ability to customize scaffold forms enables patient-specific implants and therapies. Thus, 3D printing of biopolymer hydrogels provides a versatile, scalable, and highly customizable method for creating scaffolds that support tissue growth, offering significant potential in regenerative medicine and biomedical applications. Yoo and colleagues [51] created a hybrid matrix made up of nanofibrils and hydrogels to give contact sites and differentiation signals to cells grown in 3D settings. This hybrid matrix enabled cells to detect both fibril structures and a water-rich environment that mimicked cells in the ECM. The mechanical characteristics and mass erosion of the cell matrix enabled the researchers to examine how nanofibrils affect cell matrix construction. Morphological changes in cells were also examined in order to assess the impact of nanofibrils on cell attachment and stretching [52].

### 4.4. Electrospinning Method

The electrospinning method is a well-established process for creating biopolymer hydrogel-based scaffold materials, especially in tissue engineering and regenerative medicine. This approach is well known for its capacity to produce fibrous scaffolds with nanoscale to microscale fibers that closely resemble the structure of the natural ECM, creating an optimal environment for cell attachment, development, and differentiation [53].

In electrospinning, a high-voltage electric field is applied to a polymer solution, causing the polymer jet to stretch and harden as it moves toward a collector. To improve the mechanical characteristics and water retention of biopolymer hydrogel scaffolds, natural polymers such as alginate, chitosan, gelatin, or hyaluronic acid are dissolved in appropriate solvents, frequently in conjunction with crosslinking agents or stabilizers [54,55]. The polymer solution is injected into a syringe, and a voltage is supplied between the needle and the collecting plate. As the voltage rises, electrostatic repulsion forces the polymer solution to form a fine jet, which is collected as fibers on the plate. The resultant electrospun scaffold is generally made up of ultra-fine fibers with a large surface area and porosity, both of which are required for cell penetration and nutrient exchange [56,57]. The scaffold’s fiber diameter, porosity, and alignment may be accurately controlled by altering factors such as polymer concentration, solution viscosity, applied voltage, and needle-to-collector distance. This level of control enables the creation of scaffolds with specific mechanical qualities, such as stiffness or flexibility, which are essential for simulating the mechanical environment of various tissues [58].

Crosslinking techniques (chemical or physical) can be used after construction to improve the characteristics of electrospun hydrogel scaffolds. Chemical crosslinking, such as glutaraldehyde or ionic crosslinking for alginate, can be used to stabilize fibers, increase mechanical strength, and regulate degradation rates [59]. Furthermore, electrospinning technology enables the integration of bioactive compounds (such as growth factors, medicines, or cells) into the scaffold during the production process, hence increasing the scaffold’s regeneration potential. By encapsulating bioactive compounds, the scaffold can offer targeted, controlled release, aiding in tissue repair and regeneration [60,61]. Therefore, electrospinning is a powerful technique for fabricating biopolymer hydrogel-based scaffolds due to its ability to create highly porous, fibrous structures with tunable properties. These scaffolds, with their high surface area, customizable fiber orientation, and potential for bioactive molecule incorporation, are ideal for a variety of applications in tissue engineering, wound healing, and drug delivery.

## 5. Biopolymer Hydrogel-Based Scaffold Materials for Drug Delivery Applications

Biopolymer hydrogel-based scaffold materials are rapidly being investigated for drug delivery applications because of their biocompatibility, biodegradability, and ability to enable controlled, localized release of therapeutic drugs [62]. These hydrogels, composed of natural polymers such as alginate, chitosan, gelatin, and hyaluronic acid, provide an adaptable substrate for drug encapsulation and release [63]. Their capacity to absorb vast volumes of water enables the continuous release of drugs over long periods, decreasing the need for frequent administration. The porosity and swelling behavior of biopolymer hydrogels may be controlled by varying crosslinking density, polymer concentration, and external environmental variables [64]. This parameter enables us to customize the hydrogel’s drug release kinetics, ensuring that pharmaceuticals are delivered at the proper rate and duration. Furthermore, the hydrophilic character of these materials allows for effective solubilization and encapsulation of hydrophilic drugs [65]. Furthermore, biopolymer hydrogels may be functionalized with bioactive molecules like peptides, antibodies, or growth factors to target specific tissues or cells, hence improving therapeutic effectiveness. pH-sensitive hydrogels, for example, can release their payload in reaction to changes in the local environment, such as the acidic pH of a tumor site or inflammation [66]. Therefore, biopolymer hydrogel-based scaffolds offer a promising, adaptable approach for targeted, controlled drug delivery, improving therapeutic outcomes while minimizing side effects.

### 5.1. Sustained Release Systems

Biopolymer hydrogel-based scaffold materials are gaining popularity for long-term drug delivery applications because of their biocompatibility, biodegradability, and ability to provide controlled, localized, and extended release of therapeutic compounds [67]. Because of their high water retention capacity, these hydrogels generated from natural polymers such as alginate, chitosan, gelatin, hyaluronic acid, and collagen make an excellent platform for drug encapsulation, particularly for hydrophilic drugs [68].

**Figure 6:**
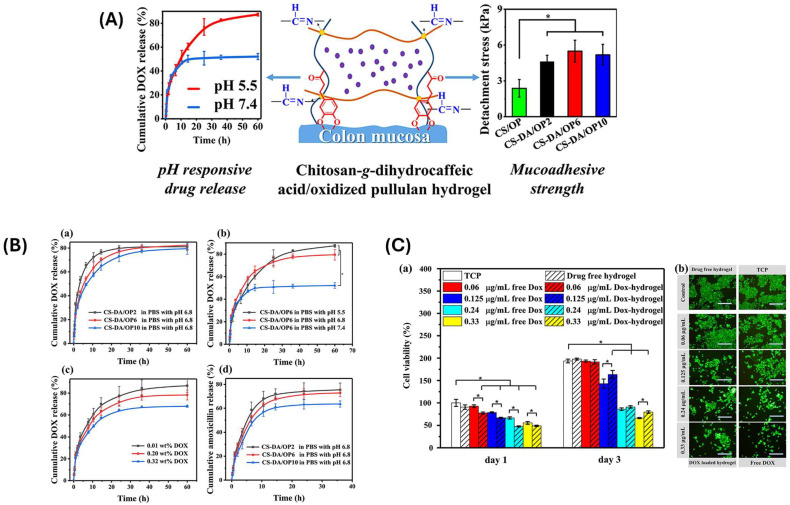
(**A**) Schematic representation of chitosan-g-dihydro caffeic acid/oxidized pullulan hydrogel. (**B**) In vitro drug release from CS-DA/OP hydrogels at 37 °C; (**a**) at pH 6.8; (**b**) at different pH values (5.5, 6.8, and 7.4); and (**c**) at pH 6.8 (3.2 mg DOX/mL hydrogel, 2.0 mg DOX/mL hydrogel, and 0.1 mg DOX/mL hydrogel). (**d**) In vitro release of amoxicillin from different CS-DA/OP hydrogels in PBS at pH 6.8. (**C**) (**a**) HCT116 cell proliferation in the drug-free CS-DA/OP6 hydrogel, DOX-loaded CS-DA/OP6 hydrogels, free DOX, and TCP on day 1 and day 3. * *p*-value < 0.0001. (**b**) Live/dead staining of HCT116 cells after being cultured for 3 days. Adapted with permission from Ref. [71]. Copyright 2019, Elsevier.

The fundamental benefit of employing biopolymer hydrogels for long-term drug administration is their capacity to manage the release kinetics of encapsulated drugs. The release rate may be controlled by altering the crosslinking density, polymer content, and hydrogel composition. Higher crosslinking densities often result in slower drug release rates because the polymer chains are more closely linked, limiting drug diffusion. In contrast, lower crosslinking densities enable faster drug release [69]. Furthermore, the swelling behavior of hydrogels has a substantial impact on drug release since it can affect drug diffusion rates. Swellable hydrogels may absorb water in the body, swell, and gradually release their payload in a regulated manner [70]. Han and colleagues [71] created a variety of multifunctional injectable hydrogels with pH-sensitive drug carriers by combining chitosan-grafted dihydro caffeic acid and oxidized pullulan (OP). The chemical structures, injectability, gelation duration, rheological characteristics, in vitro pH-dependent equilibrated swelling ratios, morphologies, and adhesive strength of the hydrogels were investigated. The drug release studies were carried out under various pH settings with doxorubicin as the model drug (Figure 6).

The breakdown rate of biopolymer hydrogels can also be adjusted to correspond to the drug release profile. A hydrogel, for example, can be made to dissolve at a regulated rate in response to tissue regeneration or healing processes, ensuring that the drug is supplied in the appropriate period [72]. This is especially effective for chronic illnesses, wound healing, and tissue engineering, which require long-term therapy. Biopolymer hydrogels can be tailored to improve drug delivery accuracy [73]. For example, pH-sensitive hydrogels can release therapeutic payloads in response to changes in local pH, such as in a tumor’s acidic environment or during inflammation [74,75]. Similarly, temperature-sensitive hydrogels may release pharmaceuticals in response to body temperature changes, making them ideal for injectable drug delivery systems [76].

### 5.2. Targeted Delivery System

Biopolymer hydrogel-based scaffold materials show great promise for targeted drug delivery since they can encapsulate therapeutic ingredients and release them in a controlled and localized way. These hydrogels, which are made up of natural polymers like alginate, chitosan, gelatin, and hyaluronic acid, have various benefits for targeted distribution, including biocompatibility, biodegradability, and the ability to be functionalized for precise targeting [77].

One of the primary advantages of biopolymer hydrogels is their capacity to encapsulate a wide range of therapeutic agents, both hydrophilic and hydrophobic, resulting in the effective delivery of diverse treatments such as small molecules, proteins, peptides, and nucleic acids [78]. Hydrogel porosity and swelling behavior may be controlled by changing factors such as polymer content and crosslinking density. These properties enable precise control over the encapsulated pharmaceuticals’ release kinetics, which can be further improved by integrating stimulus-responsive components within the hydrogel structure [79,80].

To enable targeted drug delivery, biopolymer hydrogels can be functionalized with particular ligands, antibodies, or peptides that identify and bind to cell surface receptors or antigens located on sick tissues, such as tumors or inflamed regions [81]. For example, tumor-targeting hydrogels can be functionalized with ligands such as folic acid or aptamers that preferentially attach to cancer cells, guaranteeing that the drug is delivered exclusively at the tumor location and limiting negative effects on healthy tissues [82]. Furthermore, pH-sensitive hydrogels can be developed to release drugs in reaction to the acidic environment found in tumors or inflamed tissues, increasing therapeutic specificity and efficacy [83].

Moreover, thermoresponsive hydrogels may be injected in liquid form and gelled at body temperature, providing a minimally invasive way of delivering drugs directly to the target region [84]. The disintegration rate of these hydrogels may also be controlled to guarantee that the drug is administered over time, by the body’s healing process or therapeutic requirements. Thus, biopolymer hydrogel-based scaffolds offer excellent potential for targeted drug delivery. By combining their biocompatibility, tunable release properties, and the ability to incorporate targeting ligands, these hydrogels provide an effective, localized, and controlled drug delivery system, improving therapeutic outcomes while minimizing systemic side effects [85,86].

### 5.3. Thermoresponsive and pH-Responsive Hydrogels

**Figure 7:**
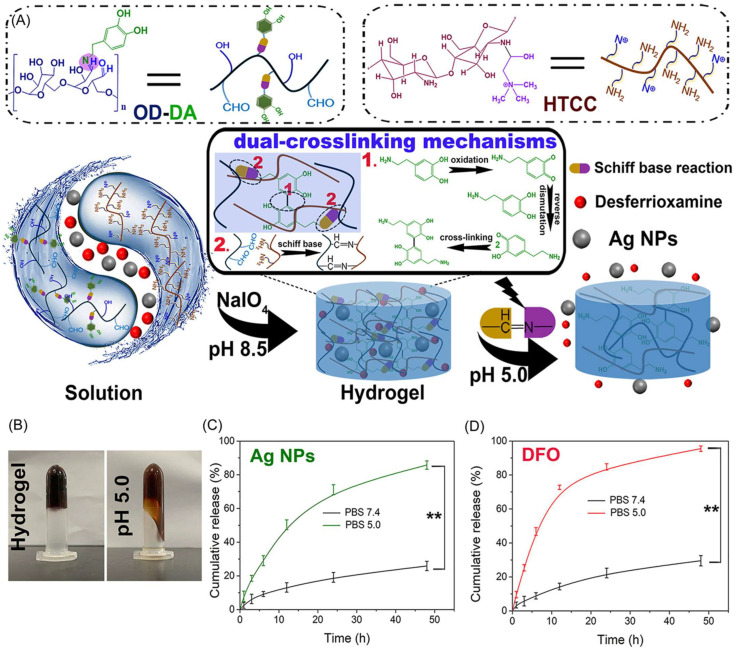
(**A**) The mechanism of the pH-responsive hydrogel. (**B**) The pH-responsive behavior of the hydrogel. Photographs of hydrogel incubated with phosphate-buffered saline (PBS) (pH 5.0) for 6 h (**right**). (**C**) The cumulative release profile of silver nanoparticles (Ag NPs), and (**D**) deferoxamine (DFO), from hydrogel@AgNPs and DFO. Adapted with permission from Ref. [88]. Copyright 2021, Elsevier. ** *p* < 0.001.

Biopolymer hydrogel-based scaffold materials have received a lot of interest for their thermoresponsive and pH-responsive drug delivery applications, thanks to their capacity to release therapeutic molecules in reaction to changes in environmental conditions. These responsive hydrogels provide considerable benefits for regulated, targeted, and localized drug administration, making them suitable for a wide range of biological applications such as cancer therapy, wound healing, and tissue engineering [87]. Hu et al. [88] developed a pH-responsive injectable multifunctional hydrogel using a mildly acidic milieu of chronically infected wounds. At chronic wound sites, pH-responsive hydrogels rapidly released silver nanoparticles (Ag NPs) to successfully kill germs, while the medication deferoxamine (DFO) stimulated the remodeling of new blood vessels, speeding up the healing of chronic skin wounds (Figure 7).

Thermoresponsive hydrogels are primarily composed of natural biopolymers such as gelatin, chitosan, or hyaluronic acid, which change phase in response to temperature variations. These hydrogels are frequently constructed to remain liquid at low temperatures and solidify when exposed to body temperature (about 37 °C). This feature enables less invasive administration, such as injectable formulations that turn into a gel once within the body [89]. Thermoresponsive hydrogels can contain drugs and gradually release them when temperatures change, causing the gel to disintegrate or expand. For example, gelatin-based hydrogels display thermoresponsive activity, allowing for continuous drug release at body temperature, which is especially useful for targeted drug delivery to tissues that require long-term therapy [90,91].

pH-responsive hydrogels, on the other hand, may release drug payloads in reaction to pH changes, making them especially valuable for targeting specific parts of the body, such as tumors or inflammatory tissues [92]. Tumor microenvironments are often more acidic than normal tissues; therefore, pH-sensitive hydrogels can selectively release drugs at the tumor location [93]. Biopolymers such as alginate, chitosan, and poly(acrylic acid) can be programmed to respond to pH changes. For example, chitosan-based hydrogels can expand and break down in acidic conditions, releasing the encapsulated drugs when exposed to the low pH seen in tumors or ulcerated tissue [94,95]. Both thermoresponsive and pH-responsive hydrogels can be combined to improve therapeutic delivery control. For example, a hydrogel that reacts to temperature and pH can provide dual stimuli for tailored drug release, enhancing therapeutic accuracy and reducing adverse effects [96].

## 6. Biopolymer Hydrogel-Based Scaffold Materials for Tissue Engineering Applications

Biopolymer hydrogel-based scaffold materials are widely used in tissue engineering due to their biocompatibility, biodegradability, and ability to replicate the ECM [97]. These hydrogels, made from natural polymers including alginate, chitosan, gelatin, and hyaluronic acid, promote cell adhesion, proliferation, and differentiation, making them excellent for tissue regeneration [98].

The basic objective of tissue engineering is to develop scaffolds that encourage cellular infiltration and the development of new tissue. Biopolymer hydrogels are particularly well suited for this because they can absorb large volumes of water, allowing for nutrition and waste transport, which is critical for cell viability [99,100].

**Figure 8:**
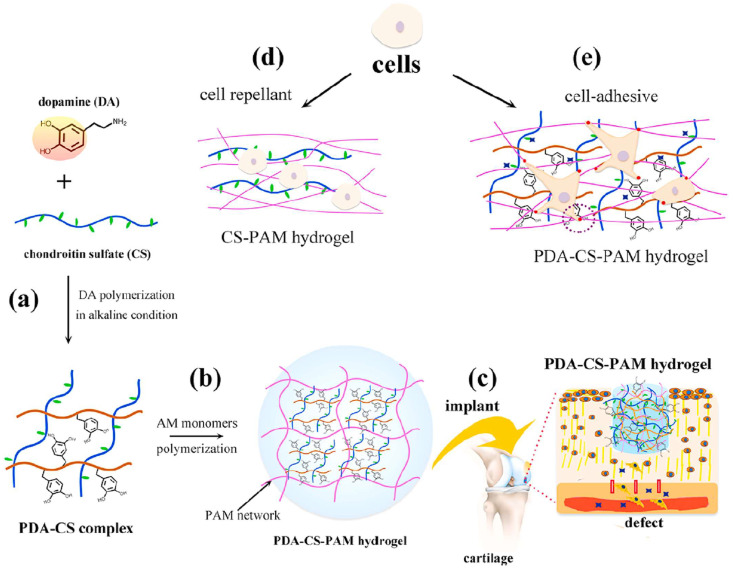
Schematic of CS-containing hydrogel for cartilage tissue engineering. (**a**) CS was complexed with polydopamine (PDA). (**b**) CS was encapsulated in a PAM (polyacrylamide) hydrogel. (**c**) The CS-containing hydrogel was implanted in a cartilage defect. (**d**,**e**) show that CS and polydopamine promoted cell adhesion on the hydrogel surfaces. Adapted with permission from Ref. [106]. Copyright 2018, American Chemical Society.

The porosity and swelling behavior of these hydrogels may be fine-tuned by altering the polymer concentration and crosslinking density, allowing for the production of scaffolds with regulated mechanical characteristics and degradation rates customized to the specific tissue under engineering [101]. These hydrogels can also be modified with bioactive compounds, such as growth factors or peptides, to improve cell signaling and accelerate tissue regeneration [102,103]. Because of their similarity to collagen, gelatin-based hydrogels are widely employed for skin and cartilage regeneration. Furthermore, biopolymer hydrogels may be directly mixed with cells during scaffold manufacturing, allowing for the development of cell-laden scaffolds that promote more successful tissue regeneration [104,105]. Lu et al. [106] developed a polydopamine (PDA) polymer that interacted with CS through non-covalent bonds to form a PDA–CS complex. Acrylamide (AM) monomers were mixed into the solution of the PDA–CS complex and polymerized to form a solid, self-standing, and adhesive PDA–CS–PAM hydrogel (Figure 8). The prepared hydrogel showed good mechanical properties. In addition, the complex was enriched with catechol groups, which provided the hydrogel with cell affinity and tissue adhesiveness for supporting cell attachment, spreading, and proliferation (Figure 8).

In tissue engineering, biopolymer hydrogel scaffolds provide a supportive environment for cell growth and tissue regeneration. Key applications are discussed in the following.

### 6.1. Cartilage and Bone Regeneration

Biopolymer hydrogel-based scaffold materials have received a lot of interest in recent years for their potential uses in cartilage and bone regeneration. These materials are extremely promising because of their biocompatibility, biodegradability, and adjustable mechanical characteristics, which are required to imitate the natural ECM of bone and cartilage tissues [107]. Hydrogels, which are three-dimensional networks of hydrophilic polymers, have a high water content, allowing them to closely mimic the hydrated environment seen in natural cartilage and bone [108]. Biopolymer-based hydrogels may be made from natural ingredients such as collagen, chitosan, alginate, and hyaluronic acid. These natural biopolymers have several benefits, including less toxicity and improved cell adhesion, both of which are essential for tissue regeneration [109,110].

**Figure 9:**
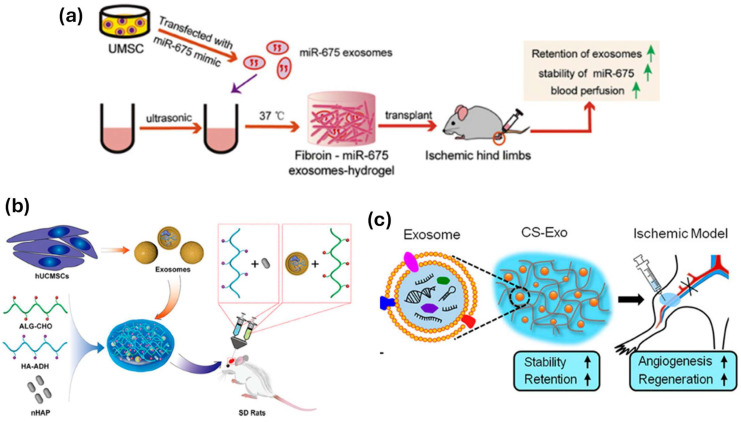
(**a**) Schematic illustration of the miR-675-loaded exosome–silk fibroin hydrogel system for age-induced vascular dysfunction treatment. Reproduced from Ref. [115], Elsevier, 2019. (**b**) Schematic illustration of the exosome–hyaluronic acid–alginate hydrogel system for bone regeneration. Reproduced with permission from Ref. [116], American Chemical Society, 2020. (**c**) Schematic illustration of the exosome–chitosan hydrogel system for muscle regeneration. Reproduced with permission from Ref. [117], American Chemical Society, 2018.

Hydrogels act as scaffolds in cartilage regeneration, providing structural support while simultaneously promoting chondrocyte proliferation and differentiation. Hydrogels may be designed to imitate cartilage mechanical characteristics, such as elasticity and stiffness, by altering the polymer concentration and crosslink density [111,112]. Collagen-based hydrogels, for example, are extensively researched for cartilage regeneration due to their inherent capacity to promote chondrocyte function. Hydrogels can include growth factors such as TGF-β and BMPs to stimulate chondrogenesis and speed up cartilage repair [113]. Liu and colleagues [114] implanted stem cell-derived exosomes in a photoinduced imine crosslinked hydrogel made from the reaction of aldehyde groups produced by light irradiation of o-nitrobenzyl alcohol moiety-modified HA and amino groups dispersed on gelatin. The exosome–hydrogel patch preserved exosomes in defective areas and effectively integrated them into native cartilage. It demonstrated high biocompatibility and was utilized as a scaffold for cartilage defect repair (Figure 9a) [115]. Yang et al. [116] effectively incorporated stem cell-derived exosomes in an injectable, hydroxyapatite-embedded, and in situ crosslinked HA–alginate composite hydrogel system (Figure 9b). Their exosome–hydrogel technology may considerably improve bone repair. Zhang et al. [117] integrated MSC-derived exosomes into an injectable chitosan hydrogel matrix that may retain exosomes at damaged locations (Figure 9c).

Bone regeneration, on the other hand, necessitates scaffolds with greater mechanical strength and the capacity to promote osteogenesis. Hydrogels composed of biopolymers such as chitosan or alginate are frequently mixed with inorganic materials such as hydroxyapatite or bioactive glass to improve osteoconductivity and mechanical qualities [118]. These composite hydrogel scaffolds stimulate osteoblast development and mineralization, which promotes bone production. Hydrogels can also be created with porosity to allow for cell infiltration and vascularization, both of which are necessary for tissue integration and healing during bone regeneration [119].

One notable benefit of biopolymer hydrogel-based scaffolds is their capacity to contain bioactive compounds and growth factors that aid in tissue healing. The regulated release of these chemicals from the hydrogel matrix can create a long-term healing environment for cartilage and bone tissues [120]. Furthermore, these hydrogels may be developed with stimulus-responsive characteristics, which allow for changes in reaction to environmental conditions such as pH or temperature, providing dynamic support for tissue regeneration over time [121].

### 6.2. Wound Healing

Biopolymer hydrogel-based scaffold materials have been developed as a novel method of wound healing, providing a potential alternative for improving tissue repair and regeneration. These materials are especially useful because of their high water content, biocompatibility, biodegradability, and ability to offer a favorable environment for cell development and migration [122]. Hydrogels are three-dimensional networks of hydrophilic polymers that can hold large quantities of water, replicating the natural ECM of tissues, which is essential for wound healing [123].

Hydrogels are moisture-retentive dressings that promote tissue regeneration by keeping the wound bed moistened. Hydration is crucial because it stimulates cell migration, alleviates pain, and prevents the development of scabs and scar tissue [124,125]. The hydrogel matrix also serves as a protective barrier, concealing the site from external pollutants while allowing for gas exchange, therefore avoiding infection and speeding up healing [126].

Biopolymers including alginate, chitosan, collagen, and hyaluronic acid are widely utilized as the foundation for hydrogel compositions in wound treatment. These natural polymers are biocompatible, which encourages cell attachment and tissue integration [127]. For example, chitosan, produced from the chitin found in crustacean shells, has antibacterial characteristics that aid in the prevention of infection in chronic or polluted wounds [128]. Similarly, collagen-based hydrogels are extensively explored since collagen is the most abundant protein in the ECM and is required for the development of new tissue throughout the healing process [129].

**Figure 10:**
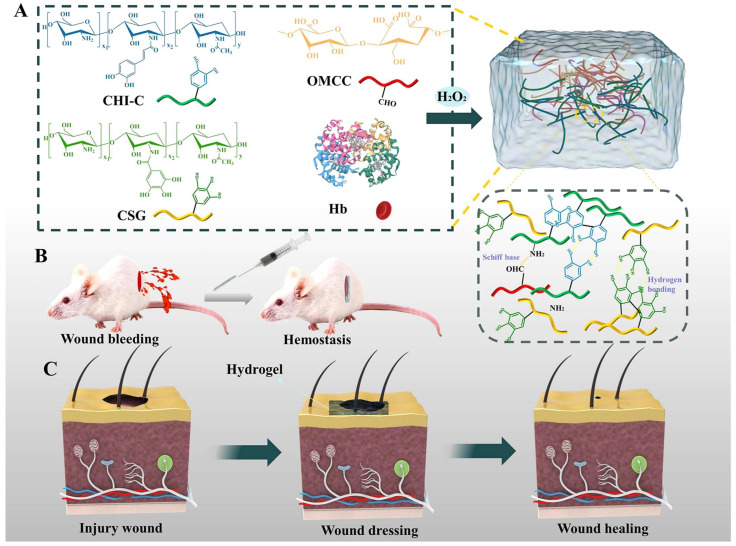
Design strategy and related characterization of hydrogels. (**A**) Schematic diagram of CHI-C/CSG/OMCC composite hydrogel preparation. (**B**) Schematic diagram of hydrogel used for wound hemostasis. (**C**) Schematic diagram of hydrogel used for wound healing. Adapted with permission from Ref. [130]. Copyright 2024, Elsevier.

Wang et al. [130] created a new ternary composite hydrogel known as caffeic acid-grafted chitosan/chitosan-coupled gallic acid/oxidized microcrystalline cellulose (CHI-C/CSG/OMCC) blood-responsive hydrogel. Beyond its well-known antibacterial and hemostatic effects, OMCC has the unusual capacity to liberate hemoglobin from red blood cells when in contact. The peroxidase-like activity of hemoglobin in the circulation catalyzes the crosslinking of CHI-C and CSG, resulting in the development of a strong hydrogel network (Figure 10).

In addition to their structural benefits, biopolymer hydrogels may be designed to release bioactive agents that aid in healing. Growth factors including epidermal growth factor (EGF), platelet-derived growth factor (PDGF), and vascular endothelial growth factor (VEGF) can be incorporated into hydrogel matrices to promote cell proliferation, angiogenesis, and tissue regeneration [131,132]. Furthermore, these hydrogels may be constructed to release these molecules in a regulated manner, ensuring that the therapeutic impact lasts over time [133,134]. Hydrogels have the added benefit of being adjustable to suit a variety of wound types, including acute, chronic, and diabetic wounds [135,136]. Hydrogel scaffolds may be created to fulfill the unique demands of distinct wound healing stages, including inflammation and tissue remodeling, by altering parameters such as polymer content, crosslinking density, and the integration of bioactive substances [137,138,139].

### 6.3. Soft Tissue

Biopolymer hydrogel-based scaffold materials have received a lot of interest for soft tissue regeneration applications because of their unique features, such as biocompatibility, biodegradability, and ability to replicate soft tissues’ native ECM. Soft tissues, such as skin, muscles, tendons, and ligaments, contain complex structures and mechanical qualities that are required for proper function. Hydrogels, which are three-dimensional networks of hydrophilic polymers, may precisely replicate the hydrated, gel-like environment present in these tissues, making them excellent candidates for soft tissue healing [140,141].

One of the primary benefits of biopolymer hydrogels is their capacity to retain a significant quantity of water, which is critical for sustaining the physiological conditions required for cell survival, proliferation, and differentiation [142]. Hydrogels made from natural biopolymers including collagen, hyaluronic acid, alginate, and fibrin are very useful for soft tissue regeneration because they promote cellular activities such as adhesion, migration, and differentiation [143,144]. Collagen-based hydrogels, for example, are commonly employed for soft tissue regeneration since collagen is an important component of many soft tissues’ extracellular matrix. Its inherent capacity to promote cell adhesion and development aids in tissue repair [145].

In soft tissue healing, hydrogels serve as scaffolds, providing structural support as well as a favorable environment for cellular activity. These scaffolds allow for regulated cell infiltration, nutrition diffusion, and waste disposal, all of which are necessary for tissue regeneration [146]. Furthermore, hydrogels may be created to have precise mechanical properties, such as elasticity and stiffness, that correspond to the features of the target tissue. Hydrogels for muscle regeneration, for example, may be more elastic to simulate muscle tissue stretchability, but those for tendon or ligament repair may have a higher tensile strength to sustain mechanical stress [100].

One distinguishing aspect of biopolymer hydrogel scaffolds is their capacity to release bioactive compounds or growth factors in a regulated manner. Encapsulating TGF-β and VEGF within the hydrogel matrix promotes cell differentiation, angiogenesis, and tissue remodeling. Hydrogel scaffolds that incorporate these growth factors can speed soft tissue regeneration and enhance functional recovery [147]. Furthermore, biopolymer hydrogels may be programmed to respond to stimuli such as pH, temperature, or mechanical stress. This flexibility improves their effectiveness at many stages of soft tissue repair, including inflammation and tissue maturation [148].

## 7. Challenges and Future Directions

Biopolymer hydrogel-based scaffold materials have shown great promise in regenerative medicine and tissue engineering due to their biocompatibility, biodegradability, and ability to imitate the natural ECM. These hydrogels have many uses, including wound healing, cartilage and bone regeneration, and soft tissue repair. Despite their promise, various hurdles must be overcome before reaching their full potential in therapeutic settings.

### 7.1. Challenges

Many biopolymer hydrogels, such as those made from collagen or hyaluronic acid, are inherently soft and flexible, making them excellent for skin and cartilage regeneration. However, they may lack the mechanical strength needed for more rigid structures like bones or tendons. The aim is to improve the mechanical characteristics of these hydrogels without sacrificing their biocompatibility. To increase strength, synthetic polymers or inorganic materials are frequently used; nevertheless, matching these features with natural biopolymer behavior remains a challenge. Controlling the breakdown rate is significantly difficult when employing biopolymer hydrogel scaffolds [149]. The scaffold must disintegrate at a pace that corresponds to tissue regeneration, ensuring that it offers enough support for tissue creation while not interfering with the healing process. However, the breakdown rate of natural polymers like collagen or chitosan can be unpredictable, resulting in scaffolds that disintegrate too rapidly or too slowly, impairing tissue regeneration. Achieving precise control over deterioration remains a significant difficulty in hydrogel creation.

While there are several manufacturing approaches for biopolymer hydrogels, scaling up production for clinical usage offers obstacles. It is challenging to achieve repeatability and uniformity in large-scale production while retaining the appropriate mechanical characteristics and bioactivity. Methods such as freeze-drying, solvent casting, and 3D printing show promise, but they may not always give the degree of control required for large-scale manufacturing without incurring considerable expenses or complicated procedures. While hydrogels promote cell proliferation, attaining enough cell infiltration, particularly in thicker tissues, remains challenging. Hydrogels frequently fail to stimulate enough vascularization (the creation of blood vessels) in bigger tissue structures [150]. This constraint may impede the transfer of oxygen and nutrients to cells within the scaffold, affecting tissue regeneration. Improving the vascularization potential of hydrogel scaffolds is important for their effectiveness in bigger and more sophisticated tissue repair scenarios.

### 7.2. Future Directions

Future research will most likely focus on creating “smart” hydrogels that respond to external stimuli like pH, temperature, and mechanical stress. These hydrogels might change their characteristics to aid tissue regeneration at various phases of healing, such as stiffening during early tissue development and disintegrating quicker after regeneration is complete. Another interesting approach is to combine natural biopolymers with synthetic or bioactive ingredients. Hybrid hydrogels can provide increased mechanical strength, regulated degradation rates, and the potential to transfer growth factors or other therapeutic substances, therefore overcoming the limits of natural hydrogels. The introduction of 3D bioprinting technology opens up intriguing possibilities for creating bespoke hydrogel scaffolds with intricate designs that imitate real tissue structures. This method allows for exact control over pore size, shape, and the integration of numerous cell types or growth factors, considerably increasing the regeneration potential of hydrogel scaffolds.

## 8. Conclusions

Biopolymer hydrogel-based scaffold materials have emerged as a viable alternative for tissue engineering and regenerative medicine due to their unique qualities such as biocompatibility, biodegradability, and ability to replicate the natural ECM. These materials provide a highly hydrated, supportive environment that encourages cell growth, migration, and differentiation, making them ideal for a variety of applications including wound healing and cartilage, bone, and soft tissue regeneration. Despite its benefits, the creation of biopolymer hydrogel-based scaffolds confronts several hurdles. These include striking the appropriate balance between mechanical strength and flexibility, regulating degradation rates to match tissue regeneration, and assuring large-scale manufacturability without sacrificing scaffold quality. Additionally, increasing appropriate vascularization and enhancing cell infiltration in thicker tissues remain significant challenges for effective therapeutic applications.

The future of biopolymer hydrogel-based scaffolds depends on overcoming these obstacles through novel techniques. Advances in hybrid materials, which blend natural and synthetic polymers, have the potential to increase hydrogel mechanical characteristics and degradation rates. Stimulus-responsive hydrogels that respond to the dynamic environment of tissue healing show potential for improving scaffold functioning. Furthermore, 3D bioprinting technology opens up new opportunities for developing bespoke scaffolds with complex topologies that closely mimic natural tissues. Overall, biopolymer hydrogel-based scaffold materials are a strong tool in the area of regenerative medicine, with the potential to transform tissue repair and regeneration. As research focuses on current difficulties, these materials are anticipated to play an increasingly crucial role in offering effective, patient-specific therapies for a wide range of tissue abnormalities and injuries. The continued development of more modern materials and manufacturing procedures will enable them to reach their full potential, resulting in more effective and broad clinical applications in the near future.

## Figures and Tables

**Table 1:** Biopolymers with their origin and chemical structures.

Biopolymer	Sources	Chemical Structure	Refs.
Chitin	Corals, lamp shells, sponges, squid, and cuttlefish	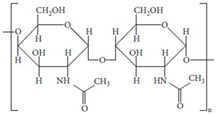	[18,19]
Chitosan	Fungi, algae, mollusks, crustaceans, and insects	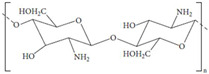	[20,21]
Alginate	Seaweed	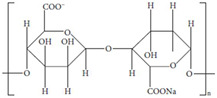	[22]
Cellulose	Seaweed, rice husk, and sugarcane bagasse, wood, bamboo, sugarbeet, banana rachis, potato tubers, cotton, hemp, coconut, grass, wheat, rice, and barley	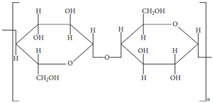	[23,24]
Starch	Potatoes, maize, cassava, rice, banana, wheat	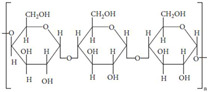	[25,26]

## Data Availability

No new data were created or analyzed in this study.
